# Successful Case of Resection of the Knee Joint, with Remarks, and a Table of Most of the Resections of the Knee Performed to the Present Time

**Published:** 1858-07

**Authors:** Daniel Brainard

**Affiliations:** Professor of Surgery in the Medical College of Chicago, etc.


THE CHICAGO

MEDICAL JOURNAL.

VOL. I. JULY, 1858. No. 7.

### ORIGINAL COMMUNICATIONS.

ARTICLE I.

### SUCCESSFUL CASE OF RESECTION OF THE KNEE JOINT, WITH REMARKS, AND A TABLE OF MOST OF THE RESECTIONS OF THE KNEE PERFORMED TO THE PRESENT TIME.

BY DANIEL BRAINARD, M. D.,
Professor of Surgery in the Medical College of Chicago, etc.

CASE. Walter Lawler, aged ten years, to the College Clinic for operation, January 20th, 1858.

*History.* About a year and a half previous to this time, he began to complain of pain in the knee. He received a severe injury, the date of which is uncertain, but it seems to have greatly aggravated the disease of the articulation, as it swelled, suppurated, and was opened in December, 1857.

*Present state.* Leg flexed. There are several openings into the knee joint, one of which in front is of large size. There is a copious discharge of serous pus. The boy is much emaciated, affected with chills, night sweats and diarrhœa, and has an anxious and suffering expression.

The hygienic condition of the patient had been as bad as could well be conceived. Improper and insufficient food, thin clothing, bad air and exposure to cold, had been constant. As the city authorities of Chicago make no provision for taking care of the poorest class in hospitals, he was necessarily continued in the same unfavorable circumstances after the operation.

The operation determined on was resection of the condyles of the femur, and dissection of the articular surface of the tibia. The reasons for this determination may be gathered from the cases and operations hereto appended.

*Operation.* Two transverse incisions were made across the joint, one above and the other below the patella, which was involved in the disease. The ligaments were divided; the end of the bone turned forward, and sawed off with the common saw of the amputating case. The exact length of the piece removed was two and a half inches. The surface of the tibia was then cut away with bone nippers; a part of it was still covered with articular cartilage, but the greater part was denuded of its cartilage, and presented a red, rough, fungous appearance, like that observed in the disease called fungus articuli by English authors.

No ligatures were required for bleeding vessels. The edges of the wound were brought together with stitches, and the member placed on a straight carved splint. The patient was under the influence of chloroform during the whole of the operation. This, in regard to mechanical difficulties in its performance, the important parts involved, and its effect on the constitution, is somewhat less serious than might be expected.

The internal surface of the joint presented the appearances already described upon the tibia. The disease had originated in the synovial membrane.

*Result.* I have not any minutes of the subsequent treatment of the case. The suppuration was very copious, and the wound showed little disposition to heal; stimulating washes were thrown into the wound; tonics, and as good a diet as could be commanded for him, were directed to be used.

At the present time, four months after the operation, the wound is entirely healed, except some small openings. There is little tenderness, and the boy is able to walk about on crutches, and his health is improving. There is still some movement at the knee, and there seems every reason to expect a useful limb.

*Table of Cases of Resection of the Knee Joint performed from* 1781 *to* 1830.

| Surgeons. | No. of Cases. | Results. |
|---|---|---|
| MM. Park | 2 | 1 cure, 1 death. |
| Filkin | 1 | Cure. |
| Moreau | 3 | 1 cure, 1 death from the operation, 1 death from dysentery when the member was almost well. |
| Mulder | 1 | Cured of the operation, died from tetanus after accouchement. |
| Fricke | 4 | 1 cure, 3 deaths. |
| Texter | 2 | Both died. |
| Jaeger | 1 | Cure. |
| Rowe | 1 | Died. |
| Crampton | 2 | 1 cure, the other recovered from the operation, but was not cured. |
| Syme | 2 | 1 cure, 1 death. |

| | |
|---|---|
| Deaths | 10 |
| Recoveries | 9 |
| Total | 19 |

*Table of Operations performed subsequent to* 1840.

| Surgeons. | Sex and Age. | Date of Operation. | Results, State of Member, Observations. |
|---|---|---|---|
| Mr. Fergusson | Male, 21 yrs | 20th July, 1850 | Death—from the operation. |
| Jones | Female, 25 " | 19th Jan., 1851 | Cure—perfect use of the member. |
| Jones | Male, 11 " | 27th April, 1851 | Ditto ditto |
| Jones | Female, 30 " | 4th Sept., 1851 | Death—of dysentery epidemic. |
| Jones | Female, 7 " | 25th Jan., 1852 | Cure—perfect use of the member. |
| Page | Male, 14 " | 7th June, 1852 | Ditto ditto |
| Jones | Male, 20 " | 25th Sept., 1852 | Ditto ditto |
| Fergusson | Female, 21 " | 30th Oct., 1852 | Ditto ditto |
| Mackenzie | Male, 42 " | 5th Feb., 1853 | Ditto ditto |
| Pritchard | Male, 20 " | 16th M'rch, 1853 | Ditto ditto |
| Thomas | Male, 12 " | 28th M'rch, 1853 | Ditto ditto |
| Fergusson | Female, 28 " | 2d April, 1853 | Death—of pyemia 60 days after oper'n |
| Jones | Male, 9 " | 17th April, 1853 | Cure—perfect use of the member. |
| Mackenzie | Male, 28 " | 5th May, 1853 | Ditto ditto |
| Cotton | Male, 9½ " | 5th Oct., 1853 | Cure—with use of member for walk'g |
| Gore | Male, 14 " | 31st Oct., 1853 | Cure—with perfect use of member. |
| Thomas | Male, 16 " | 15th Nov., 1853 | In treatment—cure. |
| Keith | Male, 9 " | 26th Nov., 1853 | Cure—with perfect use of member. |
| Mackenzie | Male, 18 " | 24th Dec., 1853 | Death—24 days after operation, which was followed by obstinate diarrhœa. |
| Stewart | ——— | ...... | Result "encouraging." |
| Butcher | Male, 33 " | 20th Jan., 1854 | Cure—perfect use of the member. |
| Ericksen | Male, 7 " | 15th Feb., 1854 | Cure—anchylosis perfect, use of mem. |
| Pemberton | Male, 12 " | 8th Feb., 1854 | Cure—perfect use of member. |
| Mackenzie | Male, 12 " | 15th April, 1854 | Death—of phthisis 12 days after oper. |
| Keith | Male, 14½ " | 17th May, 1854 | Cure—perfect use of member. |
| Jones | Female, 16 " | —— July, 1854 | In treatment, rapid progress to cure. |
| Fergusson | Male, 10 " | 29th July, 1854 | In treatment—cure rapid. |
| Holt | Male, 8 " | 7th Aug., 1854 | Progressing rapidly to cure—after six months, bone anchylosed. |
| Statham | Female, 20 " | 28th Aug., 1854 | Cure rapid—union of bone complete. |

| | | | | |
|---|---|---|---|---|
| Smith | Male, | 6 yrs. | 18th Oct., 1854. | Cure rapid—union between the bones very advanced. |
| Ericksen | Male, | 6 " | 11th Oct., 1854. | Cure rapid—good anchylosis, almost completely solid. |
| Pemberton | Male, | 12 " | 20th Dec., 1853. | Cure rapid—ligamentous union, useful member. |
| Brotherton | Male, | 10 " | 19th May, 1854. | Cure rapid—anchylosis, walks without a crutch. |
| Brotherton | Male, | 11 " | 12th Jan., 1855. | Cure rapid—useful member. |
| A surg'n of B'h army, | | Name, etc. not given. | | Died. |
| Mr. Jones | Female, | 9 yrs. | 16th Aug., 1855. | Cure—walks well. |
| Cotton | Male, | 9 " | 27th Sept., 1854. | Cure—walks well, considerable force of flexion. |
| Buck | | | | Cure—good member—anchylosis. |
| Brainard | Male, | | | Cure. |

NOTE.—A great part of the above table is translated from the *Gazette Medicale*, for 1854.

The operation of resection of the knee joint is said to have been first proposed and performed by Park in 1781. From that time to 1830 the operation was performed nineteen times. Ten of these operations resulted fatally, and nine survived the operation, although but seven seem to have been cured. Mr. Syme, in 1830, condemned the practice, and for ten years it seems not to have been performed. It was revived by Mr. Fergusson in 1850. From that time to the present we have reports of thirty-eight cases, of which thirty-two seem to have resulted favorably, and six only fatally. Of these latter, even two are supposed to have died of dysentery or diarrhœa, and one of phthisis pulmonalis.

In view of such results, we cannot but be surprised that amputation of the thigh is still generally resorted to in caries of the knee joint. In the shoulder, the elbow, the wrist, and the ankle, resection is generally preferred to amputation, both in wounds and caries. In regard to the hip and knee, the question may be regarded as at present, *sub judice*.

The points which should determine the preference are the following:

1. Is it more dangerous than amputation of the thigh?
2. Is the member more useful after recovery than an artificial one?

The facts speak for themselves on the first point, and there can be little doubt that resection of the knee is less dangerous than amputation of the thigh.

It would appear also that little doubt could exist on the second head, although much must depend in this respect on the facility of procuring artificial members of a perfect construction; yet we find, so late as 1855, Mr. Syme was opposed to the operation, and it is to his authority that the reluctance to perform it is in a measure due. After the above cases have been contemplated, it surely can no longer be considered a "curious experiment," as Mr. Syme styled it.

There is, however, a distinction to be drawn. It will be noticed, for example, that the fatal results were, with one exception, among the oldest class of patients. I believe, too, that but one of these cases was performed for a wound, and this proved fatal. The extent to which the removal of the ends of bone may be carried has not been determined. Two and a half inches seems about the average, but in some cases more was removed.

Anchylosis is the ordinary result of the operation, and yet very good limbs have been obtained with considerable mobility. Unless the destruction of bone is very extensive, amputation of the thigh should never be performed for scrofulous disease of the knee. If any operation be required, resection should be resorted to.

